# Characteristics and survival of patients with gynecological cancers who refuse radiotherapy: a retrospective cohort study

**DOI:** 10.1186/s12905-023-02720-6

**Published:** 2023-11-01

**Authors:** Shuangli Zhang, Jie He, Jun Liu

**Affiliations:** 1 Department of Gynecology, Beijing Ditan Hospital, Capital Medical University, No.8 Jingshun East Street, Chaoyang District, Beijing, 100200 China; 2 Department of Gynecology, Beijing Liangxiang Hospital, Capital Medical University, Beijing, 102401 China

**Keywords:** Gynecological cancers, Refusal of radiotherapy, Overall survival, Stage

## Abstract

**Background:**

Radiotherapy improves survival for many cancer patients. However, some patients still refuse radiotherapy despite the recommendations of their physicians. We aimed to investigate the impact of refusing recommended radiotherapy on overall survival in patients with gynecological cancers (GC) and attempted to describe what characteristics are associated with the refusal of radiotherapy.

**Methods:**

Data were extracted from the Surveillance, Epidemiology and End Result (SEER) database for patients who were diagnosed with GC and recommended for radiotherapy between 1988 and 2016. Kaplan–Meier and multivariate Cox regression analyses were utilized to analyze the impact of refusal of radiotherapy on overall survival. Univariate and multivariate logistic regression analyses were used to identify characteristics associated with refusal of radiotherapy.

**Results:**

In total, 1,226 of 208,093 patients (0.6%) refused radiotherapy. Multivariate Cox regression analysis showed that refusal of radiotherapy was associated with poorer overall survival in GC patients with stage I/II [hazard ratio (HR) = 1.64; 95% confidence interval (CI), 1.50–1.79], but may not affect overall survival in patients with stage III/IV (HR = 1.03; 95%CI, 0.84–1.25). Multivariate logistic regression analysis demonstrated that factors such as older age (40–65 years, > 65 years), unmarried status (divorced, single, widowed), higher foreign-born rate (1.87-2.82%, 1.51–2.19), refusal of surgery (recommended but not performed), and higher grade (poorly differentiated, undifferentiated/anaplastic) may increase the likelihood of refusing radiotherapy (all *P* < 0.05). Factors that may reduce the likelihood of refusing radiotherapy include higher income (> 42,810$), lower grade (well-differentiated), primary site of ovarian cancer, and no/unknown chemotherapy (all *P* < 0.05).

**Conclusion:**

Refusal of radiotherapy is related to worse overall survival in GC patients with stage I/II, and many characteristics may affect a patient’s choice of refusal of radiotherapy.

**Supplementary Information:**

The online version contains supplementary material available at 10.1186/s12905-023-02720-6.

## Background

Gynecological cancers (GC) are cancers that affect the ovaries, uterus, cervix, vulva, and vagina [1], and they are the second most common cancers in women after breast cancer [2]. In 2020, there were an estimated 981,234 new diagnoses of GC, including 604,127 cervical cancers, 313,959 ovarian cancers, 45,240 vulvar cancers, and 17,908 vaginal cancers [2]. In addition, there were 574,505 new deaths from GC worldwide [2]. Most early-stage GC, such as cervical, vulvar, and endometrial cancers, can be cured with surgery, or in combination with adjuvant radiotherapy or chemotherapy [3–5]. However, approximately 20–80% of cancer patients experience cancer recurrence after adequate treatment [6, 7]. Radiotherapy was used in isolated patients with local recurrence based on disease location and prior treatments.

Radiotherapy plays a central role in the treatment of GC, especially for some cancers that can also be cured with radiotherapy alone [8, 9]. Radiotherapy in combination with surgery and systemic therapy plays an important role in improving patient local control and prolonging overall survival of patients [10]. Despite these benefits of radiotherapy, it is not uncommon for patients to refuse radiotherapy in clinical practice. Aizer et al. indicated that about 0.9% of cancer patients refused radiotherapy despite the recommendations of their physicians [11]. Parsons et al. found that 5.9% of patients with endometrial cancer refused radiotherapy [12]. Importantly, the refusal of radiotherapy has been reported to be associated with poor survival outcomes in several cancers [13–15]. For example, refusal of radiotherapy has been found to be related to worse survival in patients with esophageal adenocarcinoma [14]. The study by Schwam et al. showed that head and neck cancer patients who refused postoperative radiotherapy had a significantly lower 3-year overall survival rate than patients who received postoperative radiotherapy [15]. However, few studies have reported the effect of refusal of radiotherapy on the survival of GC patients. Furthermore, there is limited evidence to assess characteristics associated with refusal of radiotherapy when recommended by the physician.

This study aimed to investigate the impact of refusal of recommended radiotherapy on overall survival of patients with GC based on a large national database. In addition, we attempted to describe what characteristics are associated with the refusal of radiotherapy.

## Methods

### Data source and patients

Data used in this retrospective cohort study were extracted from the Surveillance, Epidemiology and End Result (SEER)-18 registries (Nov 2020 Sub) database between 1988 and 2016. SEER database covers approximately 28% of the United State population and collects information on cancer statistics to reduce the cancer burden (https://seer.cancer.gov/). Patients diagnosed with GC were identified using the International Classification of Diseases for Oncology, version 3 (ICD-3) codes, including cervical cancer (C530-539), ovarian cancer (C569), vaginal cancer (C529), vulvar cancer (C510-519) and endometrial cancer (C540-549, C559). Patients who met the following inclusion criteria were included: (1) patients with pathological diagnosis of primary GC; (2) patients’ age ≥ 18 years; (3) patients with only one primary tumor; (4) patients received radiotherapy or radiotherapy was recommended but not performed due to the refusal of patients; (5) patients with complete data on marital status, tumor stage, and surgery, etc. Patients diagnosed with GC by autopsy or death certificate were excluded. Because this study used de-identified patient information from a public database (SEER database), this study was exempted from the Institutional Review Board of Beijing Ditan Hospital, Capital Medical University. The need for written informed consent was waived by the Institutional Review Board of Beijing Ditan Hospital, Capital Medical University due to retrospective nature of the study. All methods were performed in accordance with the relevant guidelines and regulations.

### Variables

The primary outcomes of this study were overall survival and refusal of radiotherapy. Patients were followed up until 2017, and follow-up ended if the patient died during this period. Patient information including age (< 40, 40–65, and > 65 years), race (white, black, and others), marital status at diagnosis (divorced, married, separated, single, and widowed), residence (rural and urban), proxy measures (country-level data) of socioeconomic status [family income (< 36,580, 36,580–42,810, and > 42,810 $), unemployed (percentage of individuals with unemployed in a county; <52.4%, 52.4–71.4%, and > 71.4%), education (percentage of individuals with less that high-school education in a county; <18.75%, 18.75–28.20%, and > 28.20%), language isolation (percentage of people with English-language difficulties; <1.87%, 1.87–2.82%, and > 2.82%), foreign born (percentage of foreigners born in a county)], surgery (yes, recommended but not performed, and not performed), grade (well differentiated, moderately differentiated, poorly differentiated, and undifferentiated/anaplastic), primary site (cervical cancer, uterine cancer, ovarian cancer, and vulval cancer), chemotherapy (yes and no/unknown), the American Joint Committee on Cancer (AJCC) stage (I/II and III/IV), and overall survival status (survival and death) were collected.

### Statistical analysis

Categorical variables were expressed as numbers and percentages [n (%)], and the Chi-square test (χ^2^) was used for comparison between groups. Characteristics of GC patients were described according to patients who received radiotherapy and those who refused radiotherapy. Kaplan-Meier (KM) survival curves and log-rank test or two-stage test were performed to calculate the survival time for patients who refused radiotherapy. Variables with *P* < 0.05 in the comparison of the characteristics of patients who received radiotherapy and those who refused radiotherapy were included in the multivariate Cox regression and logistic regression analyses for adjustment. Multivariate Cox regression analysis was utilized to assess the association between refusal of radiotherapy and overall survival, and reported as hazard ratio (HR) and 95% confidence interval (CI). Then, univariate- and multivariate logistic regression analyses were used to identify factors associated with refusal of radiotherapy, and presented as odds ratio (OR) and 95%CI. All statistical tests were two-tailed, and *P*-value < 0.05 was defined as significant. Statistical analyses were performed using the SAS 9.4 software (SAS Institute Inc., Cary, NC, USA) and RStudio 4.0.3 software.

## Results

### Characteristics of patients

**Fig. 1:**
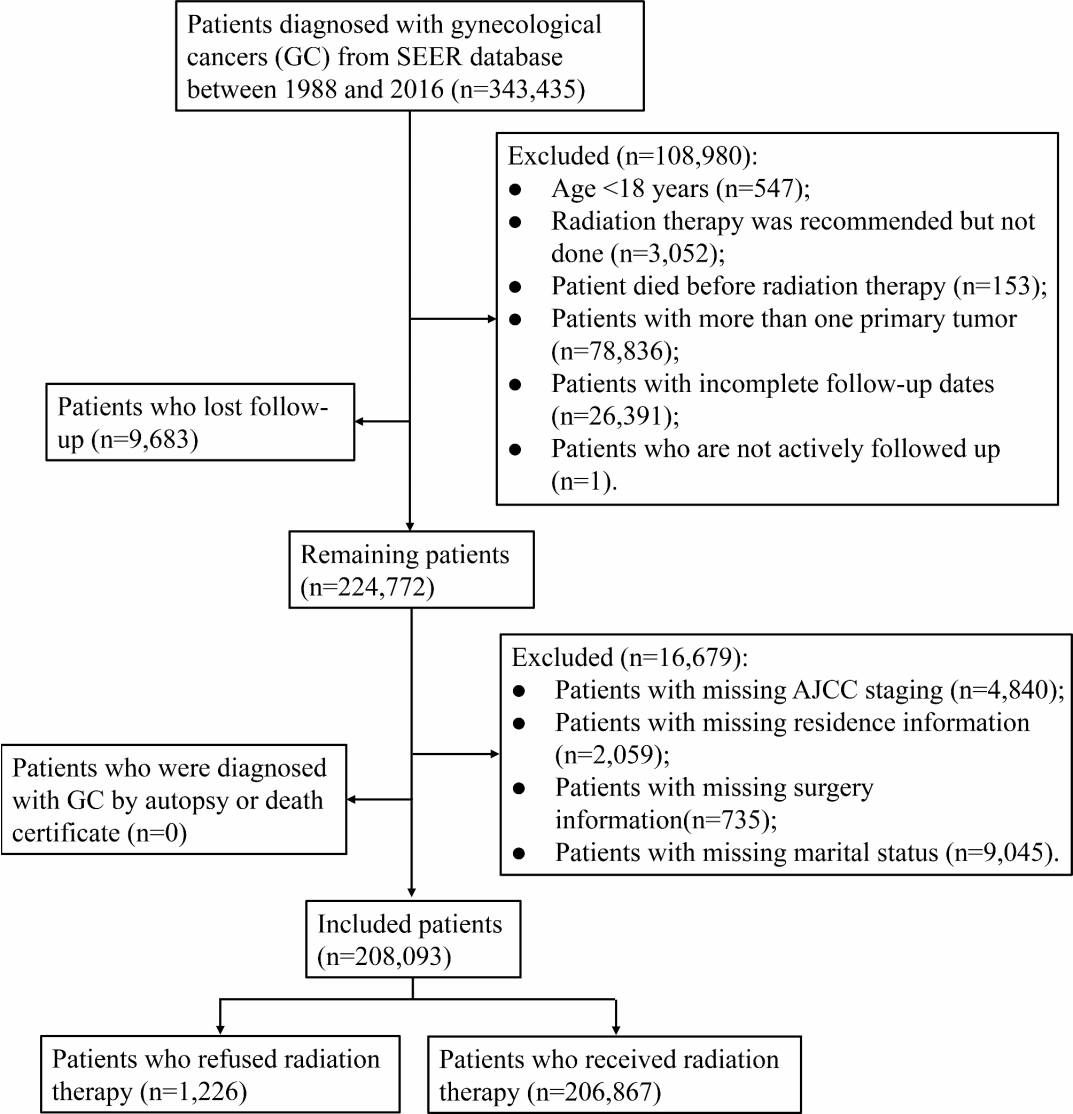
Flow chart for selection of patients

**Fig. 2:**
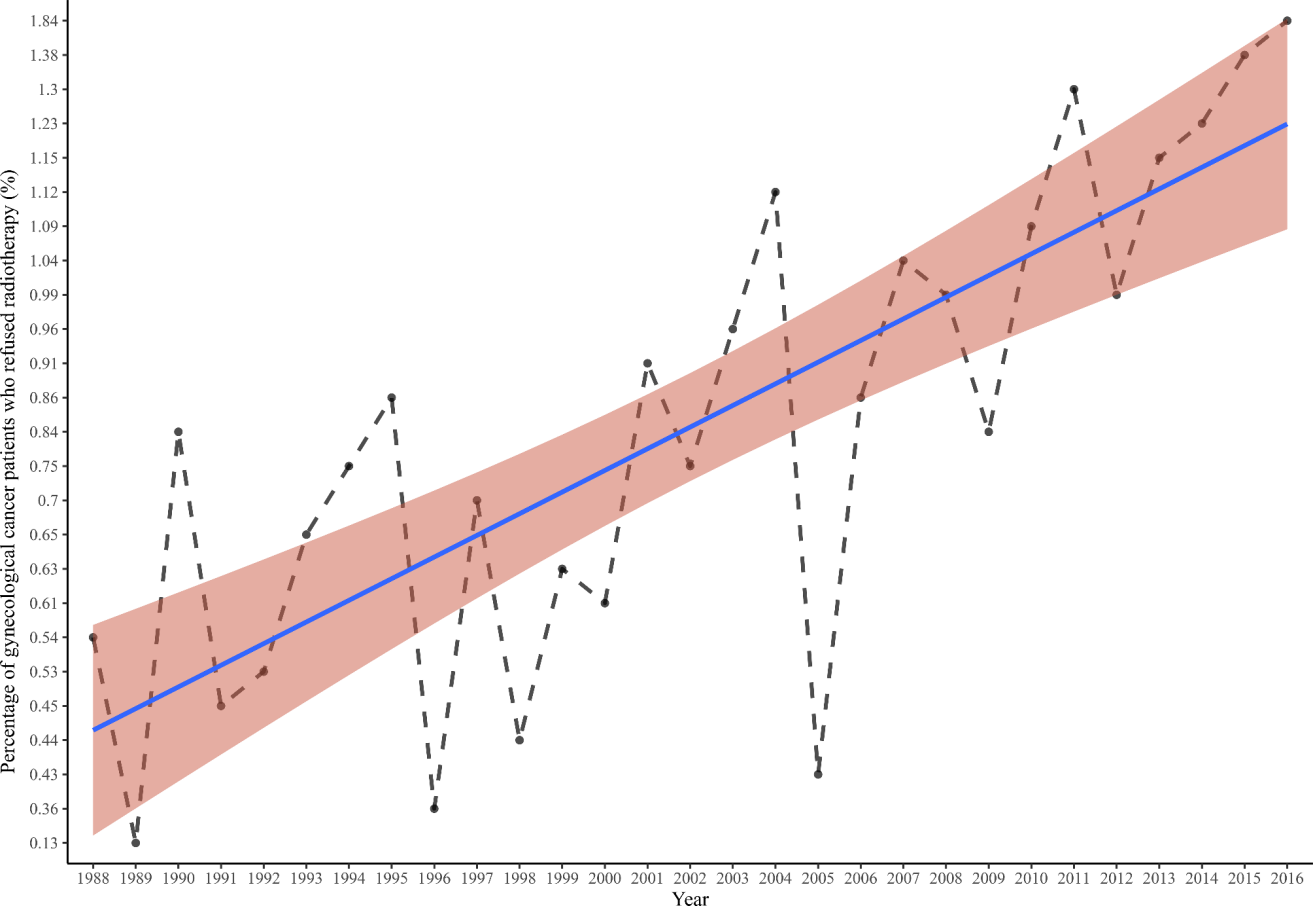
Trends in the percentage of gynecological cancers (GC) patients who refused radiotherapy from 1988 to 2016

A total of 343,435 patients diagnosed with GC were identified from the SEER database between 1988 and 2016. After screening, 135,342 patients were excluded, and 208,093 patients who met the inclusion criteria were included in the analysis (Fig. 1). Table 1 presents the characteristics of all included patients. Of the 208,093 patients, 118,284 (56.8%) were 40–65 years, 169,831 (81.6%) were whites, and 109,802 (52.8%) were married. In the terms of county-level data, 69,269 (33.3%) patients had an income of 36,580 − 42,810$, 69,825 (33.6%) patients had a regional unemployed percentage of 52.4-71.4%, 70,021 (33.6%) patients with education level less than high school of 18.75-28.20%, and 68,648 (33.0%) patients with regional language isolation percentage of 1.87-2.82%. There were 182,462 (87.7%) patients in stage I/II and 25,631 (12.3%) patients in stage III/IV. For treatment, 186,047 (89.4%) patients underwent surgery, and 71,269 (34.2%) patients received chemotherapy. There were 206,867 (99.4%) patients received radiotherapy and 1,226 (0.6%) patients refused radiotherapy. The median follow-up time for all patients was 56.00 (21.00, 120.00) months, while the median follow-up time for patients who refused radiotherapy was 27.50 (11.00, 72.00) months. At the end of follow-up, 128,204 (61.6%) patients were alive and 79,889 (38.4%) patients had died. Figure 2 demonstrates trends in the percentage of GC patients who refused radiotherapy from 1988 to 2016. The proportion of GC patients who refused radiotherapy increased from 1988 to 2016.

**Table 1:** Baseline characteristics of gynecological cancers (GC) patients receiving and refusing radiotherapy

Variables	All (n = 208,093)	Radiation (n = 206,867)	Refuse radiation (n = 1226)	*P*
Age (years), n (%)				< 0.001
<40	21,454 (10.3%)	21,387 (10.3%)	67 (5.46%)	
40–65	118,284 (56.8%)	117,774 (56.9%)	510 (41.6%)	
>65	68,355 (32.8%)	67,706 (32.7%)	649 (52.9%)	
Race, n (%)				0.969
Black	19,843 (9.54%)	19,726 (9.54%)	117 (9.54%)	
Others	18,419 (8.85%)	18,308 (8.85%)	111 (9.05%)	
White	169,831 (81.6%)	168,833 (81.6%)	998 (81.4%)	
Marital status at diagnosis, n (%)				< 0.001
Divorced	22,284 (10.7%)	22,136 (10.7%)	148 (12.1%)	
Married	109,802 (52.8%)	109,310 (52.8%)	492 (40.1%)	
Separated	2905 (1.40%)	2885 (1.39%)	20 (1.63%)	
Single	41,561 (20.0%)	41,319 (20.0%)	242 (19.7%)	
Widowed	31,541 (15.2%)	31,217 (15.1%)	324 (26.4%)	
Residence, n (%)				0.303
Rural	154,493 (74.2%)	153,599 (74.3%)	894 (72.9%)	
Urban	53,600 (25.8%)	53,268 (25.7%)	332 (27.1%)	
Income ($), n (%)				< 0.001
<36,580	67,432 (32.4%)	66,981 (32.4%)	451 (36.8%)	
36,580–42,810	69,269 (33.3%)	68,710 (33.2%)	559 (45.6%)	
>42,810	71,392 (34.3%)	71,176 (34.4%)	216 (17.6%)	
Unemployed, n (%)				< 0.001
<52.4%	66,969 (32.2%)	66,684 (32.2%)	285 (23.2%)	
52.4-71.4%	69,825 (33.6%)	69,378 (33.5%)	447 (36.5%)	
>71.4%	71,299 (34.3%)	70,805 (34.2%)	494 (40.3%)	
Education (less than high school), n (%)				0.009
<18.75%	67,875 (32.6%)	67,522 (32.6%)	353 (28.8%)	
18.75-28.20%	70,021 (33.6%)	69,601 (33.6%)	420 (34.3%)	
>28.20%	70,197 (33.7%)	69,744 (33.7%)	453 (36.9%)	
Language isolation, n (%)				0.368
<1.87%	62,776 (30.2%)	62,390 (30.2%)	386 (31.5%)	
1.87-2.82%	74,999 (36.0%)	74,551 (36.0%)	448 (36.5%)	
>2.82%	70,318 (33.8%)	69,926 (33.8%)	392 (32.0%)	
Foreign born, n (%)				0.025
<5.04%	67,895 (32.6%)	67,535 (32.6%)	360 (29.4%)	
5.04-15.66%	68,648 (33.0%)	68,207 (33.0%)	441 (36.0%)	
>15.66%	71,550 (34.4%)	71,125 (34.4%)	425 (34.7%)	
Surgery, n (%)				< 0.001
Not Performed	18,500 (8.89%)	18,397 (8.89%)	103 (8.40%)	
Recommended but not performed	3546 (1.70%)	3430 (1.66%)	116 (9.46%)	
Yes	186,047 (89.4%)	185,040 (89.4%)	1007 (82.1%)	
Grade, n (%)				< 0.001
Well differentiated (grade I)	63,653 (30.6%)	63,409 (30.7%)	244 (19.9%)	
Moderately differentiated (grade II)	62,448 (30.0%)	62,053 (30.0%)	395 (32.2%)	
Poorly differentiated (grade III)	62,432 (30.0%)	61,982 (30.0%)	450 (36.7%)	
Undifferentiated/anaplastic (grade IV)	19,560 (9.40%)	19,423 (9.39%)	137 (11.2%)	
Primary site, n (%)				< 0.001
Cervical cancer	35,033 (16.8%)	34,801 (16.8%)	232 (18.9%)	
Endometrial cancer	119,348 (57.4%)	118,450 (57.3%)	898 (73.2%)	
Ovarian cancer	47,360 (22.8%)	47,327 (22.9%)	33 (2.69%)	
Vulvar cancer	6352 (3.05%)	6289 (3.04%)	63 (5.14%)	
Chemotherapy, n (%)				< 0.001
No/Unknown	136,824 (65.8%)	135,727 (65.6%)	1097 (89.5%)	
Yes	71,269 (34.2%)	71,140 (34.4%)	129 (10.5%)	
AJCC stage, n (%)				0.127
I/II	182,462 (87.7%)	181,405 (87.7%)	1057 (86.2%)	
III/IV	25,631 (12.3%)	25,462 (12.3%)	169 (13.8%)	
Overall survival, n (%)				< 0.001
Survival	128,204 (61.6%)	127,593 (61.7%)	611 (49.8%)	
Death	79,889 (38.4%)	79,274 (38.3%)	615 (50.2%)	

Note: AJCC, the American Joint Committee on Cancer; unemployed, percentage of individuals with unemployed in a county; education, percentage of individuals with less that high-school education in a county; language isolation, percentage of people with English-language difficulties; foreign born, percentage of foreigners born in a county

Furthermore, there were significant differences in patients who received and refused radiotherapy among age, marital status, income, unemployed, education, foreign born, surgery, grade, primary site, chemotherapy, and overall survival (all *P* < 0.05).

### Impact of refusal of radiotherapy on overall survival

**Fig. 3:**
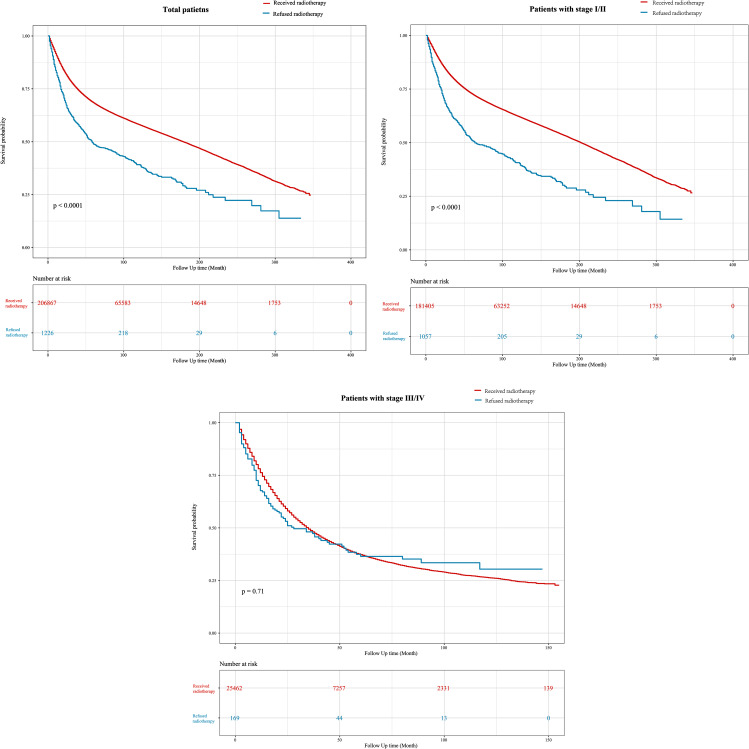
Kaplan–Meier (K-M) curves for the impact of receiving and refusing radiotherapy on the overall survival of GC patients with different stages. **(A)** total patients; **(B)** patients with stage I/II; **(C)** patients with stage III/IV

Figure 3 shows the K-M survival curves for the impact of receiving and refusing radiotherapy on the overall survival of patients with different AJCC stages. GC patients who refused radiotherapy were associated with a poorer overall survival (Fig. 3A; *P* < 0.001). In addition, patients with AJCC stage I/II who refused radiotherapy also had worse overall survival than those who received radiotherapy (Fig. 3B; *P* < 0.001). However, no significant difference in overall survival was observed in AJCC stage III/IV patients who refused radiotherapy and those who received radiotherapy (Fig. 3C; *P* = 0.71). Since the K-M curves of patients with AJCC stage III/IV crossed, a two-stage test was used to further examine the difference in overall survival of these patients. The results indicated that there was still no significant difference in overall survival among AJCC stage III/IV patients who refused radiotherapy and those who received radiotherapy (*P* = 0.337).

Table 2 presents the impact of refusal of radiotherapy on overall survival in different populations. For GC patients with different AJCC stages, refusal of radiotherapy was associated with worse overall survival in patients with AJCC stage I/II (HR = 1.64; 95%CI, 1.50–1.79), but may not affect overall survival in patients with AJCC stage III/IV (HR = 1.03; 95%CI, 0.84–1.25). For patients with different years at diagnosis, refusal of radiotherapy was related to poorer overall survival in patients with years at diagnosis < 2000 (HR = 1.48; 95%CI, 1.22–1.79), years at diagnosis 2000–2013 (HR = 1.69; 95%CI, 1.54–1.85), and years at diagnosis > 2013 (HR = 1.56; 95%CI, 1.19–2.03). In addition, refusal of radiotherapy was associated with worse overall survival in both patients who received surgery (HR = 1.57; 95%CI, 1.42–1.72) or those who did not (HR = 1.29; 95%CI, 1.11–1.49).

**Table 2:** Effect of refusal of radiotherapy on overall survival of GC patients in different populations

Populations	Variables	Multivariate analysis	
		HR (95%CI)	*P*
AJCC I/II stage (n = 182,462)	Radiotherapy	Ref	
	Refuse of radiotherapy	1.64 (1.5–1.79)	< 0.001
AJCC III/IV stage (n = 25,631)	Radiotherapy	Ref	
	Refuse of radiotherapy	1.03 (0.84–1.25)	0.806
Years at diagnosis < 2000 (n = 35,014)	Radiotherapy	Ref	
	Refuse of radiotherapy	1.48 (1.22–1.79)	< 0.001
Years at diagnosis 2000–2013 (n = 140,339)	Radiotherapy	Ref	
	Refuse of radiotherapy	1.69 (1.54–1.85)	< 0.001
Years at diagnosis > 2013 (n = 32,740)	Radiotherapy	Ref	
	Refuse of radiotherapy	1.56 (1.19–2.03)	< 0.001
No surgery (n = 22,046)	Radiotherapy	Ref	
	Refuse of radiotherapy	1.29 (1.11–1.49)	< 0.001
Surgery (n = 186,047)	Radiotherapy	Ref	
	Refuse of radiotherapy	1.57 (1.42–1.72)	< 0.001

Note: GC, gynecological cancers; AJCC, the American Joint Committee on Cancer; HR, hazard ratio; CI, confidence interval;

Multivariate Cox regression analysis adjusted for age, marital status, income, unemployed, grade, education, foreign born, surgery, primary site, and chemotherapy

### Characteristics associated with refusal of radiotherapy

Table 3 displays univariate and multivariate logistic regression analyses of factors associated with refusal of radiotherapy in AJCC stage I/II patients. Univariate analysis indicated that factors that may affect refusal of radiotherapy include age, marital status, income, unemployed, education, foreign born, surgery, grade, primary site, and chemotherapy.

**Table 3:** Univariate and multivariate logistic regression analyses of factors associated with refusal of radiotherapy in patients with stage I/II

Variables	Univariate analysis		Multivariate analysis	
	OR (95%CI)	*P*	OR (95%CI)	*P*
Age (years)				
<40	Ref		Ref	
40–65	1.43 (1.09–1.91)	0.013	1.60 (1.20–2.16)	0.002
>65	3.32 (2.54–4.43)	< 0.001	2.89 (2.15–3.96)	< 0.001
Marital status at diagnosis				
Married	Ref		Ref	
Divorced	1.62 (1.33–1.97)	< 0.001	1.45 (1.19–1.76)	< 0.001
Separated	1.51 (0.91–2.48)	0.109	1.46 (0.88–2.42)	0.142
Single	1.32 (1.12–1.57)	0.001	1.34 (1.13–1.59)	< 0.001
Widowed	2.34 (2.01–2.72)	< 0.001	1.35 (1.14–1.59)	< 0.001
Income ($)				
<36,580	Ref		Ref	
36,580–42,810	1.25 (1.09–1.42)	0.001	0.93 (0.79–1.11)	0.444
>42,810	0.45 (0.37–0.53)	< 0.001	0.26 (0.20–0.33)	< 0.001
Unemployed				
<52.4%	Ref		Ref	
52.4-71.4%	1.53 (1.30–1.80)	< 0.001	0.88 (0.74–1.05)	0.164
>71.4%	1.69 (1.45–1.99)	< 0.001	0.86 (0.67–1.10)	0.219
Education (less than high school)				
<18.75%	Ref		Ref	
18.75-28.20%	1.24 (1.06–1.44)	0.006	1.13 (0.96–1.33)	0.146
>28.20%	1.30 (1.12–1.51)	0.001	0.81 (0.64–1.03)	0.080
Foreign born				
<5.04%	Ref		Ref	
5.04-15.66%	1.19 (1.03–1.39)	0.022	1.82 (1.51–2.19)	< 0.001
>15.66%	1.10 (0.95–1.28)	0.214	1.60 (1.30–1.97)	< 0.001
Surgery				
Not Performed	Ref		Ref	
Recommended but not performed	5.91 (4.35–8.11)	< 0.001	5.52 (4.03–7.60)	< 0.001
Yes	0.95 (0.74–1.23)	0.66	1.11 (0.86–1.46)	0.439
Grade				
Moderately differentiated (grade II)	Ref		Ref	
Poorly differentiated (grade III)	1.23 (1.06–1.43)	0.006	1.73 (1.49–2.01)	< 0.001
Undifferentiated/anaplastic (grade IV)	1.28 (1.03–1.57)	0.021	2.39 (1.91–2.95)	< 0.001
Well differentiated (grade I)	0.60 (0.50–0.71)	< 0.001	0.54 (0.45–0.64)	< 0.001
Primary site, n (%)				
Cervical cancer	Ref		Ref	
Endometrial cancer	1.12 (0.95–1.32)	0.181	1.05 (0.87–1.28)	0.597
Ovarian cancer	0.11 (0.07–0.16)	< 0.001	0.13 (0.09–0.20)	< 0.001
Vulvar cancer	1.46 (1.07–1.97)	0.014	1.18 (0.85–1.61)	0.323
Chemotherapy, n (%)				
Yes	Ref		Ref	
No/Unknown	0.24 (0.19–0.29)	< 0.001	0.28 (0.22–0.35)	< 0.001

Note: OR, odds ratio; 95%CI, confidence interval

Multivariate logistic regression analysis adjusted for age, marital status, income, unemployed, grade, education, foreign born, surgery, primary site, and chemotherapy

For multivariate analysis, factors include older age [40–65 years, OR = 1.60, 95%CI, 1.20–2.16; >65 years, OR = 2.89, 95%CI, 2.15–3.96], unmarried status [divorced, OR = 1.45, 95%CI, 1.19–1.76; single, OR = 1.34, 95%CI, 1.13–1.59; widowed, OR = 1.35, 95%CI, 1.14–1.59], higher foreign born [1.87-2.82%, OR = 1.82, 95%CI, 1.51–2.19; >2.82%, OR = 1.60, 95%CI, 1.30–1.97], refused surgery [recommended but not performed, OR = 5.52, 95%CI, 4.03–7.60], and higher grade [poorly differentiated, OR = 1.73, 95%CI, 1.49–2.01; undifferentiated/anaplastic, OR = 2.39, 95%CI, 1.91–2.95] may increase the likelihood of refusing radiotherapy. Factors that may reduce the likelihood of refusing radiotherapy include higher income [> 42,810$, OR = 0.26, 95%CI, 0.20–0.33], lower grade [well differentiated, OR = 0.54, 95%CI, 0.45–0.64], primary site of ovarian cancer (OR = 0.13, 95%CI, 0.09–0.20), and no/unknown chemotherapy (OR = 0.28, 95%CI, 0.22–0.35).

## Discussion

Radiotherapy is one of the important methods in cancer treatment, especially for localized or solid cancers. However, some cancer patients may refuse radiotherapy despite the recommendations of their physicians. Our results found that the proportion of GC patients who refused radiotherapy increased from 1988 to 2016. Refusal of radiotherapy was associated with decreased overall survival in patients with AJCC stage I/II GC, but refusal of radiotherapy may not affect overall survival in patients with AJCC stage III/IV. Furthermore, factors that may increase the likelihood of refusal of radiotherapy include older age, unmarried status, higher foreign born, refusal of surgery, and higher grade. Patients with higher income, lower tumor grades, and those who did not receive chemotherapy were more likely to receive radiotherapy.

Previous studies have focused more on the effect of refusal of surgery on survival in cancer patients [16–18]. Radiotherapy is one of the three common ways of cancer treatment, and the impact of refusal of radiotherapy on patient survival needs to be concerned [19]. Our results found that patients with AJCC stage I/II GC who refused radiotherapy had a lower overall survival compared to those who received radiotherapy, while there was no difference in overall survival between the two groups in patients with AJCC stage III/IV. Aizer et al. showed that cancer-specific mortality in ovarian cancer and uterine cancer patients who refused radiotherapy was significantly higher than in patients who received radiotherapy [11]. A large sample study conducted by Parsons et al. demonstrated that refusal of radiotherapy was associated with a significantly lower 5-year overall survival in patients with endometrial cancer [12]. Hanna et al. indicated that radiotherapy can provide important and irreplaceable local control and overall survival benefits in GC patients under optimal utilization [20]. Furthermore, a meta-analysis found that the 5-year overall survival rate was 50–61% in patients with stage I/II cervical cancer who received radiotherapy only, and 24% in stage III/IV patients [9]. Our results showed a significant difference in overall survival between patients who refused radiotherapy and those who received radiotherapy in patients with stage I/II GC.

Characteristics that may be associated with refusal of radiotherapy in patients with GC were also explored in this study. Patients with older age, unmarried status, the county of higher foreign born, refused surgery, and higher tumor grade was more likely to refuse radiotherapy. Parsons et al. also found that higher age was associated with a higher likelihood of refusal of radiotherapy [12]. This phenomenon may be related to the underestimation of their survival expectations and the fear of treatment options in older cancer patients [21, 22]. Married patients were more likely to receive radiotherapy than divorced/single/widowed patients. This may be attributed to the fact that married patients may receive more social support to face the stress of receiving radiotherapy [23, 24]. Patients who refused surgery may also refuse radiotherapy, possibly because patients who refused recommended surgery may also be negative about alternative recommended treatments. In addition, our results also indicated that patients with higher income and those who did not receive chemotherapy were more likely to receive radiotherapy. Socioeconomic status has an important influence on the treatment compliance of cancer patients [25]. Patients with higher socioeconomic status are less worried about the financial burden of treatment and more likely to trust physician recommendations [26, 27]. For patients who did not receive chemotherapy, perhaps because they did not receive other treatments, they would not refuse radiotherapy, which is commonly used in cancer treatment. Patients who do not receive recommended radiotherapy may not understand the benefits of radiotherapy for survival in patients with cancers and may be distrustful of the health care system [28]. Surveys of patients and physicians revealed significant differences in their knowledge about radiotherapy, such as how radiotherapy works and whether patients are able to work during treatment [29]. In addition, precision management of GC plays an important role in patient survival due to differences in secondary and tertiary prevention of different types of GC [30]. Therefore, for patients with GC who may refuse radiotherapy (e.g., older and unmarried), clinicians may need to spend more time making patients clearly understand their condition, the recommended treatments, the benefits of the treatments, and the impact of the treatments on their daily life.

We used a large sample and nearly 30 years of data from the SEER database to explore the impact of refusal of radiotherapy on overall survival in GC patients and to analyze characteristics associated with refusal of radiotherapy. This study may provide evidence for the relationship between refusal of radiotherapy and overall survival in patients with GC. However, there are several limitations to our study. First, due to database-based study, such a database can often be associated with miscoding and missing data. Second, insurance status had a significant impact on patient treatment choices but was not included in the analysis because the relevant data in the database were not recorded until 2007. We measure the effect of insurance status by incorporating other relevant variables such as income, education level, etc. Third, there are only “yes” and “no/unknown” statuses regarding the use of chemotherapy, with unclear information about chemotherapy was recommended but refused by patients. Fourth, comorbidities are associated with overall survival, but information related to comorbidities was not available due to limitations of the SEER data. Fifth, the impact of changes in radiotherapy guidelines for GC on patient survival could not be assessed due to limitations of the SEER database.

## Conclusions

A large national database was used to investigate the impact of refusing recommended radiotherapy on overall survival in patients with GC. Refusal of radiotherapy was associated with poorer overall survival in GC patients with stage I/II, but it may not affect overall survival in patients with stage III/IV. Factors include older age, unmarried status, higher foreign born, refused surgery, and higher grades may increase the likelihood of refusal of radiotherapy. Factors that may reduce the likelihood of refusal of radiotherapy include higher income, lower grade, the primary site of ovarian cancer, and no/unknown chemotherapy.

### Electronic supplementary material

Below is the link to the electronic supplementary material.

Supplementary Material 1

## Data Availability

All data relevant to the study are included in the article is available from SEER database, https://seer.cancer.gov/. The dataset used in this study is presented in the Supplementary Material (“Raw data.xlsx”).

## Abbreviations

GC: Gynecological cancers
SEER: Surveillance, Epidemiology and End Result
ICD-3: International Classification of Diseases for Oncology, version 3
AJCC: the American Joint Committee on Cancer
KM: Kaplan-Meier
HR: Hazard ratio
CI: Confidence interval
OR: Odds ratio
